# Autologous apoptotic neutrophils inhibit inflammatory cytokine secretion by human dendritic cells, but enhance Th1 responses

**DOI:** 10.1002/2211-5463.12904

**Published:** 2020-06-29

**Authors:** Gyöngyike Emese Majai, Péter Gogolák, Márta Tóth, Judit Hodrea, Dorottya Horváth, László Fésüs, Éva Rajnavölgyi, Attila Bácsi

**Affiliations:** ^1^ Department of Biochemistry and Molecular Biology Faculty of Medicine University of Debrecen Hungary; ^2^ Department of Immunology Faculty of Medicine University of Debrecen Hungary; ^3^ Doctoral School of Molecular Cellular and Immune Biology University of Debrecen Hungary; ^4^ Doctoral School of Molecular Medicine University of Debrecen Hungary; ^5^ Present address: Division of Clinical Immunology Department of Internal Medicine Faculty of Medicine University of Debrecen Hungary; ^6^ Present address: MTA‐SE ‘Lendület’ Diabetes Research Group Hungarian Academy of Sciences and Semmelweis University Budapest Hungary

**Keywords:** autologous neutrophils, dendritic cells, phagocytosis, physiological immunity

## Abstract

Neutrophils represent the most abundant cell type in peripheral blood and exhibit a remarkably brief (6–8 h) half‐life in circulation. The fundamental role of these professional phagocytes has been established in acute inflammation, based on their potential to both initiate and receive inflammatory signals. Furthermore, neutrophils also take part in maintaining chronic inflammatory processes, such as in various autoimmune diseases. Here, we demonstrate that human autologous apoptotic neutrophils are readily engulfed by immature monocyte‐derived dendritic cells (moDCs) with similar efficiency as allogeneic apoptotic neutrophils [Majai G *et al*. (2010) J Leukoc Biol 88, 981–991]. Interestingly, in contrast to the allogeneic system, exposure of moDCs to autologous apoptotic neutrophils inhibits LPS + IFN‐γ‐induced production of inflammatory cytokines in a phagocytosis‐independent manner. Autologous apoptotic neutrophil‐primed DCs are able to modulate T‐cell responses by inducing the generation of IFN‐γ‐secreting cells while hampering that of IL‐17A‐producing cells. Our observations indicate that capture of autologous apoptotic neutrophils by immature DCs may impede further neutrophil‐mediated phagocytosis and tissue damage, and allow increased clearance of dying cells by macrophages.

## Graphical abstract

Immature human monocyte‐derived dendritic cells (moDCs) are able to engulf apoptotic autologous or allogeneic neutrophils in the same manner. Exposure of moDCs to autologous apoptotic neutrophils inhibits secretion of inflammatory cytokines parallel with an impact on T‐cell response by inhibiting IL‐17A and increasing IFN‐γ production. These contribute to the maintenance of a steady‐state condition of the immune system.

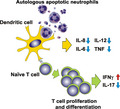

**Abbreviations**
Ab: antibody
APC: antigen‐presenting cell
DCs: dendritic cells
moDCs: monocyte‐derived dendritic cells
NET: neutrophil extracellular trap

Neutrophils represent the most abundant cell types in blood circulation due to their constant production in the bone marrow. The essential role of these cells in acute inflammation has previously been attributed to three important antimicrobial mechanisms, that is phagocytosis, degranulation and the release of extracellular traps (NETs). However, literature data also demonstrate that neutrophils possess a sophisticated repertoire of functional responses including the production of cytokines and a wide array of inflammatory factors with the potential to modulate or influence activities of other cell types, thus contributing to the development of chronic inflammatory reactions [1, 2]. In case of ongoing inflammation, the first cell types emerging are the neutrophils, which are recruited rapidly to the site of pathogen entry or tissue damage [3]. Being present in the peripheral tissues, neutrophils acquire the potential to initiate and receive signals with the capability to modulate the functional attributes of both innate and adaptive immune responses [4]. Among neutrophil‐derived mediators, alarmins are able to recruit conventional and plasmacytoid dendritic cells (DCs) as well as precursors of DCs to the site of inflammation [5]. Human monocyte‐derived DCs (moDCs) act as professional antigen‐presenting cells (APCs) and can readily be recruited to sites of infection, thus playing an essential role in linking innate and adaptive immunity through the induction of antigen‐specific T cells and having the potential to stimulate antigen‐specific MHC class II‐restricted CD4^+^ cells as well as MHC class I‐restricted CD8^+^ T‐cell responses referred to as ‘cross‐priming’ [6].

The interaction of neutrophils and moDCs is regulated either by contact‐dependent mechanism or via the action of alarmins, cytokines, inflammatory mediators and extracellular vesicles [5, 7]. A number of alarmins are released from neutrophil granules in response to exogenous and/or endogenous danger signals and able to recruit and activate professional APCs, thus initiating adaptive immune responses [5]. The engulfment of allogeneic apoptotic neutrophils by moDCs depends not only on immunogenic signals released from the dying cells, but also on the subtype of the contributing other cells which in turn might be dependent on the actual tissue environment [8]. It has been shown in our previous study that CD1a^–^ moDCs are more active in the engulfment of apoptotic neutrophils than CD1a^+^ moDCs [9]. Importantly, the engulfment of allogeneic apoptotic neutrophils has also been found to sensitize CD1a^−^ moDCs for high IL‐8, TNF‐α and IL‐6 secretion, while CD1a^+^ cells respond to allogeneic apoptotic neutrophils and additional inflammatory stimuli with elevated IL‐12 and IL‐10 production resulting in the polarization of autologous T lymphocytes to Th1‐type effector cells [9].

Various strategies with the potential to modulate neutrophil killing, phagocytosis, degranulation, oxidative burst, NET release and subsequent NET engulfment, together with their impact on moDC‐mediated activities, have already been studied [10, 11, 12]. Importantly, only limited data are available on the possible effects of human autologous apoptotic neutrophils on moDC functions and on their impact on the outcome of T‐cell responses. In this study, we used an *in vitro* phagocytic system established and optimized previously [13] for monitoring the possible effects of autologous apoptotic neutrophils on human moDCs. Here, we confirm that the phagocytosis of apoptotic neutrophils by moDCs is not affected by the autologous or allogeneic origin of the neutrophils. Exposure to autologous apoptotic neutrophils decreases inflammatory cytokine production by moDCs in a phagocytosis‐independent manner. Coculture with autologous apoptotic neutrophil‐primed moDCs enhances IFN‐γ, while it decreases IL‐17A production by autologous T cells.

## Materials and methods

### Cell culture reagents

Human peripheral blood mononuclear cells (PBMCs) were isolated from healthy blood donors by density gradient centrifugation using Ficoll Paque Plus (Amersham Biosciences, Buckinghamshire, UK) as described previously [9]. To generate immature moDCs, the freshly isolated monocytes were plated into six‐well culture dishes (Costar, Merck, Darmstadt, Germany) at a density of 2 × 10^6^ cells·mL^−1^ and cultured for 5 days in AIMV medium (Invitrogen, Carlsbad, CA, USA) containing 800 U·mL^−1^ GM‐CSF and 500 U·mL^−1^ IL‐4 (PeproTech EC, London, UK). On day 3, the same amounts of GM‐CSF and IL‐4 were added to the cell cultures. Autologous neutrophils were isolated from freshly drawn human peripheral blood samples by density gradient centrifugation using Histopaque 1119 and Histopaque 1077 (Sigma‐Aldrich, Budapest, Hungary). The cells were cultured for 16 h in IMDM (Invitrogen) supplemented with 10% human AB serum (Sigma‐Aldrich) to let the cells undergo spontaneous apoptosis. Monocyte‐depleted autologous PBMCs were kept at −70 °C in cell‐freezing medium (FBS–DMSO at 9 : 1 all from Sigma‐Aldrich).

All experiments were undertaken with the understanding and written consent of each subject and meet the standards set by the Declaration of Helsinki. The study methodologies were approved by the local ethics committee.

### Human phagocytosis assays

**Fig. 1:**
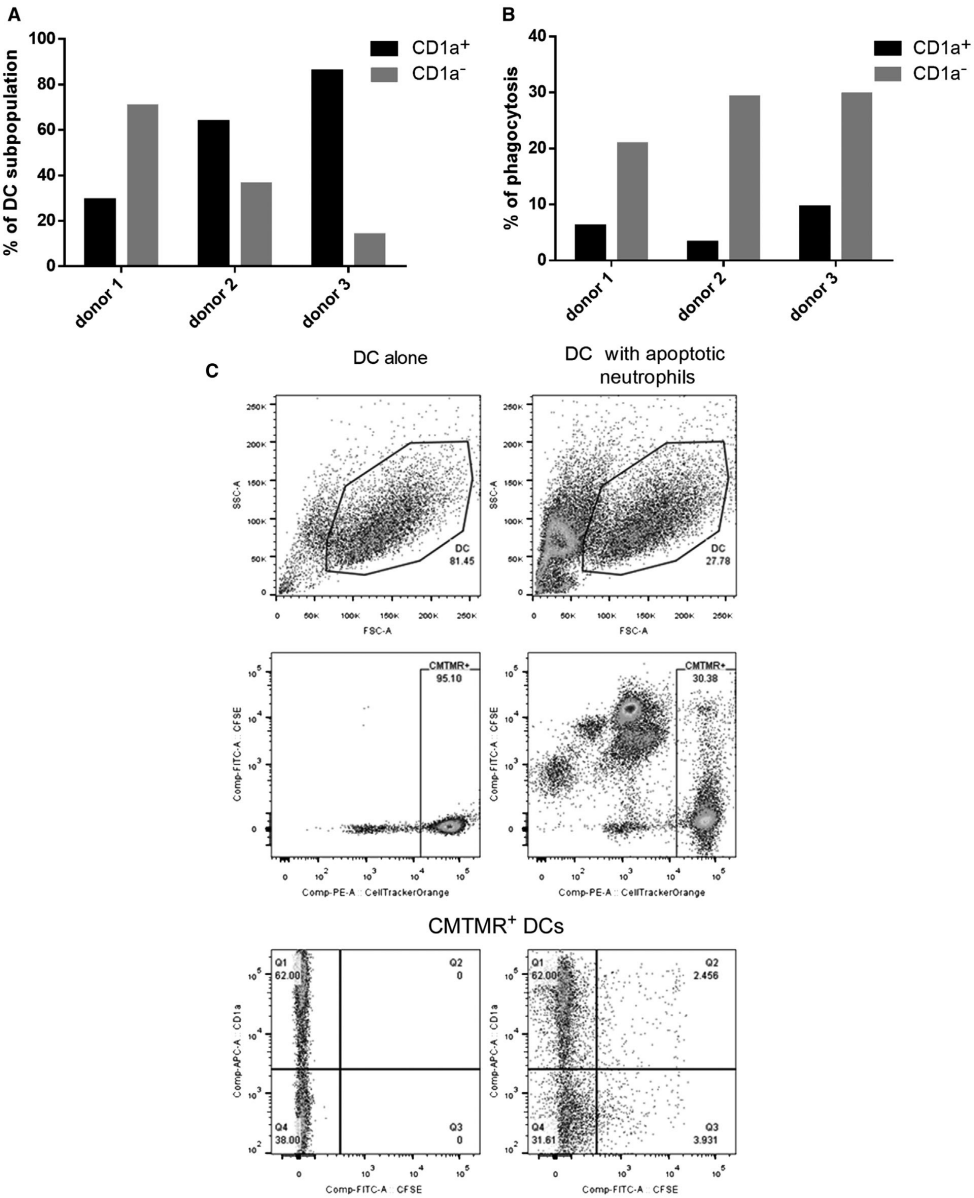
Phagocytosis of autologous apoptotic neutrophils by immature moDCs. Immature DCs harvested on day 5 were stained by CellTracker Orange CMTMR and then incubated with the yellow cell‐tracker CFDA‐SE‐labelled autologous apoptotic neutrophils at a ratio of 1 : 5 for 8 h. (A) The percentage of CD1a^+^ and CD1a^−^ moDCs was measured in 3 independent experiments (B) parallel with the determination of phagocytic activity in the above‐mentioned cell populations. Panel C shows results of a representative flow cytometric measurement. The upper dot plots display the light scatter properties of nonprimed DCs (upper left dot plot) and those of autologous apoptotic neutrophil‐primed DCs (upper right dot plot). The CMTMR‐stained DCs and the dimly fluorescent DC‐derived cell debris can be distinguished on the middle left dot plot. The CMTMR‐stained DCs and the CFDA‐SE‐labelled free apoptotic neutrophils can be clearly distinguished on the middle right dot plot. The bottom left dot plot shows the percentage of CD1a^+ ^(APC) and CD1a^−^ moDCs stained with CMTMR, while the bottom right dot plot displays a higher ratio of CD1a^−^ moDCs with increased CFDA fluorescence compared to CD1a^+^ moDCs.

Human moDCs were stained with the CellTracker Orange CMTMR ((5‐(and‐6)‐(((4‐chloromethyl)benzoyl)amino)tetramethylrhodamine) (Invitrogen), whereas the freshly isolated neutrophils were labelled with the green‐yellow fluorescent cell‐tracker dye CFDA‐SE (5‐(and‐6)‐carboxyfluorescein diacetate, succinimidyl ester) (Invitrogen) as described previously [13]. The labelled apoptotic neutrophils were washed three times with PBS, while moDCs were counted and replated in fresh medium. In some experiments, moDCs were pretreated with 15 µm cytochalasin D (CytD; Sigma‐Aldrich) for 30 min. The moDCs and the apoptotic neutrophils were cocultured for 8 h at a ratio of 1 : 5 at 37 °C in a humidified atmosphere containing 5% CO_2_. After that, moDCs were collected by trypsinization, washed repeatedly with PBS and fixed with 1% PFA in PBS (Sigma‐Aldrich). Phagocytosis was investigated by flow cytometry analysis (FACSAria III cytometer, BD Biosciences, Környe, Hungary, Immunocytometry Systems). The orange‐yellow emission of the DCs and the green‐yellow emission of the neutrophils were clearly distinguishable (Fig. 1B). DCs in the DC–apoptotic neutrophil coculture samples were gated by the help of CMTMR staining. The phagocytosis was estimated by the CFDA‐SE fluorescence of the gated DCs (indicated as CFSE on the figure plots). Cytometric data were analysed by the flowjo software (Tree Star, Ashland, OR, USA).

### Cell surface labelling

To detect CD1a and DC‐SIGN (CD209) expression, moDCs were washed in PBS supplemented with 0.5% bovine serum albumin and the cells were labelled in residual volume ice‐cold buffer for 30 min with allophycocyanin (APC)‐conjugated mouse anti‐human CD1a and fluorescein isothiocyanate (FITC)‐conjugated mouse anti‐human CD209 monoclonal antibodies (BioLegend, San Diego, CA, USA). Cell analysis was performed by a FACSCalibur or FACSAria III flow cytometers (BD Biosciences, Immunocytometry Systems).

### Determination of cytokine release

The differentiated moDCs were cocultured with autologous apoptotic neutrophils for 8 h as described above. Culture supernatants were harvested and stored at −20 °C until cytokine measurements. A fraction of the cells was washed with PBS and then treated with 0.1 μg·mL^−1^ LPS (*Escherichia coli*; Alexis Biochemicals, San Diego, CA, USA) and 10 ng·mL^−1^ IFN‐γ (PeproTech) for another 16 h, and the supernatants were collected and stored at −20 °C until cytokine measurements. The chosen LPS + IFN‐γ concentration effectively stimulated the moDC pro‐inflammatory cytokine production in previous experiments. The concentrations of IL‐8, IL‐6, TNF‐α and IL‐12p70 were measured by using the human inflammatory cytometric bead array (BD Pharmingen, Diagon, Budapest) approach.

### Human cytokine ELISPOT assays

Monocyte‐derived DCs were cocultured with nonlabelled autologous apoptotic neutrophils for 8 h followed by seeding the moDCs to 48‐well plates. Apoptotic cell‐primed moDCs were cocultured with autologous lymphocytes at a ratio of 1 : 25 for 5 days at 37 °C in a humidified atmosphere containing 5% CO_2_. On day 5, the nonadherent lymphocytes were collected and subjected to IFN‐γ and IL‐17A detection for 48 h by using ELISPOT assays (IFN‐γ – eBioscience; IL‐17A – eBioscience, San Diego, CA, USA and Mabtech AB, Cincinnati, OH, USA) on MultiScreen HTS PVDF Plates (Millipore S.A, Darmstadt, Germany). For detection of IL‐17A‐producing cells, purified anti‐human CD3 antibody (Ab, 0.5 µg·mL^−1^; BD Biosciences) together with anti‐IL‐17A capture Ab was added to the coating buffer for the mitogenic stimulation of CD3^+^ T cells. After 48 h at 37 °C, the cells were removed and the plates were washed with PBS. Detection of cytokine spots was performed by biotinylated IFN‐γ‐ or IL‐17A‐specific Ab and avidin‐HRP conjugate and colorigenic substrate mix (AEC Substrate Set; BD Biosciences). After termination of the reaction by tap water, the air‐dried plates were analysed by a computer‐assisted ELISPOT image analyser (Series 1 ImmunoSpot Analyzer, immunospot version 4.0 Software; Academic Cellular Technology).

### Statistical analysis

ANOVA multiparametric test was performed using IBM (Armonk, NY, USA) spss 25 software for statistical analysis. Differences were considered to be statistically significant at *P* < 0.05.

## Results

### Autologous apoptotic neutrophils are engulfed by immature monocyte‐derived dendritic cells

Our previous results demonstrated that immature human moDCs are able to internalize allogeneic apoptotic neutrophils. Furthermore, it has been found that CD1a^–^ moDCs exhibit increased phagocytic activity compared to CD1a^+^ moDCs [9]. To extend these findings, in this study we investigated the internalization of autologous apoptotic neutrophils by CD1a^+^ and CD1a^−^ subsets of immature moDCs. Therefore, CD1a expression on immature moDCs was measured in three independent experiments, parallel with the determination of phagocytic activity in both CD1a^+^ and CD1a^−^ cell populations. Figure 1A shows a high donor‐dependent variability in the ratio of CD1a^+^ and CD1a^−^ cells. Flow cytometry analysis revealed that immature moDCs can take up CFDA‐SE‐labelled autologous apoptotic neutrophils and also that CD1a^−^ DCs have a higher capacity to engulf autologous apoptotic cells than CD1a^+^ DCs (Fig. 1B,C). The percentage of phagocytic cells was 20–30% in the first cell population, while it was < 10% in the latter one (Fig. 1B).

### The engulfment of autologous apoptotic neutrophils inhibits LPS‐induced inflammatory cytokine responses in human moDCs

**Fig. 2:**
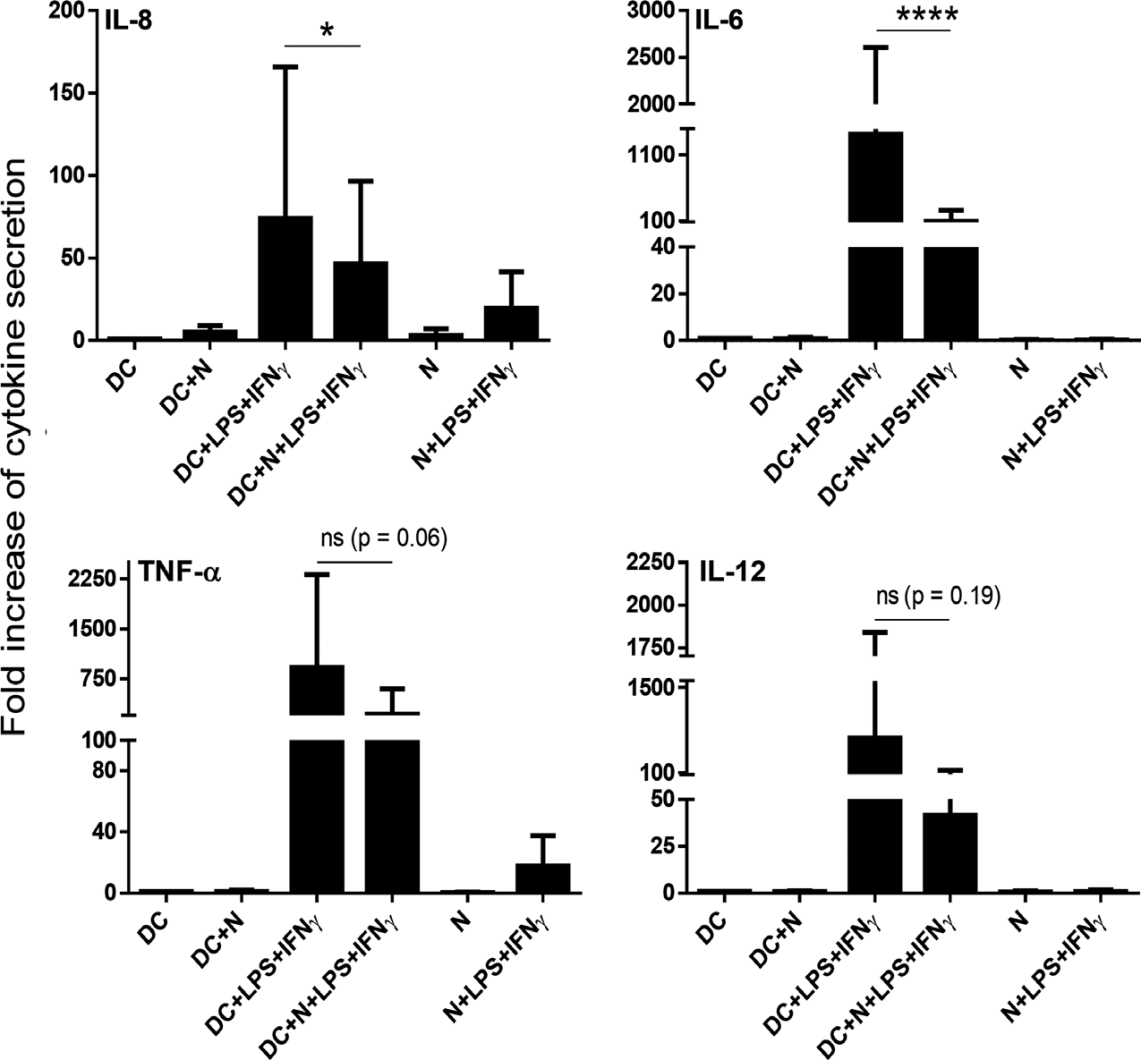
Cytokine profiling of moDCs cocultured with autologous apoptotic neutrophils. Immature moDCs were cocultured with autologous apoptotic neutrophils (N) for 8 h. A fraction of the cells was treated with 0.1 μg·mL^−1^ LPS and 10 ng·mL^−1^ IFN‐γ for another 16 h. Culture supernatants were harvested and stored at −20 °C until cytokine measurements. The concentrations of IL‐8, IL‐6, TNF‐α and IL‐12p70 were measured by using a human inflammatory cytometric bead array. Mediator secretion by treated moDCs is presented as fold increase compared to cytokine production of nonprimed moDCs. The mean ± SD values were calculated from 6 independent experiments. The basal cytokine secretion by nonprimed, immature DCs were the following: IL‐8: 993.42 ± 830.38; IL‐6: 36.67 ± 24.51; IL‐12: 1.51 ±1 .18; TNF‑α: 17.58 ± 22.66 pg·mL^−1^. ns, not significant, *P < 0.05, ****P < 0.0001. ANOVA multiparametric test was performed for statistical analysis.

Next, we studied how a prior uptake of autologous apoptotic neutrophils affects moDCs' responses to inflammatory stimuli. To do this, immature moDCs were cocultured with autologous apoptotic neutrophils for 8 h and then stimulated with LPS + IFN‐γ for an additional 16 h. In contrast to our previous observation that the preceding internalization of allogeneic apoptotic neutrophils upregulates the LPS + IFN‐γ‐induced secretion of the pro‐inflammatory cytokines, in the autologous assay system the production of IL‐8, IL‐6, TNF‐α and IL‐12 by autologous apoptotic cell‐primed moDCs showed a trend to be lower than that by nonprimed moDCs (Fig. 2). However, the differences were found to be statistically significant in case of IL‐8 and IL‐6 only (Fig. 2). These results indicate that the engulfment of autologous apoptotic neutrophils has a potential to inhibit chemokine and cytokine responses by human moDCs induced by subsequent inflammatory stimuli.

### Apoptotic autologous neutrophils inhibit inflammatory cytokine production by moDCs in a phagocytosis‐independent manner

**Fig. 3:**
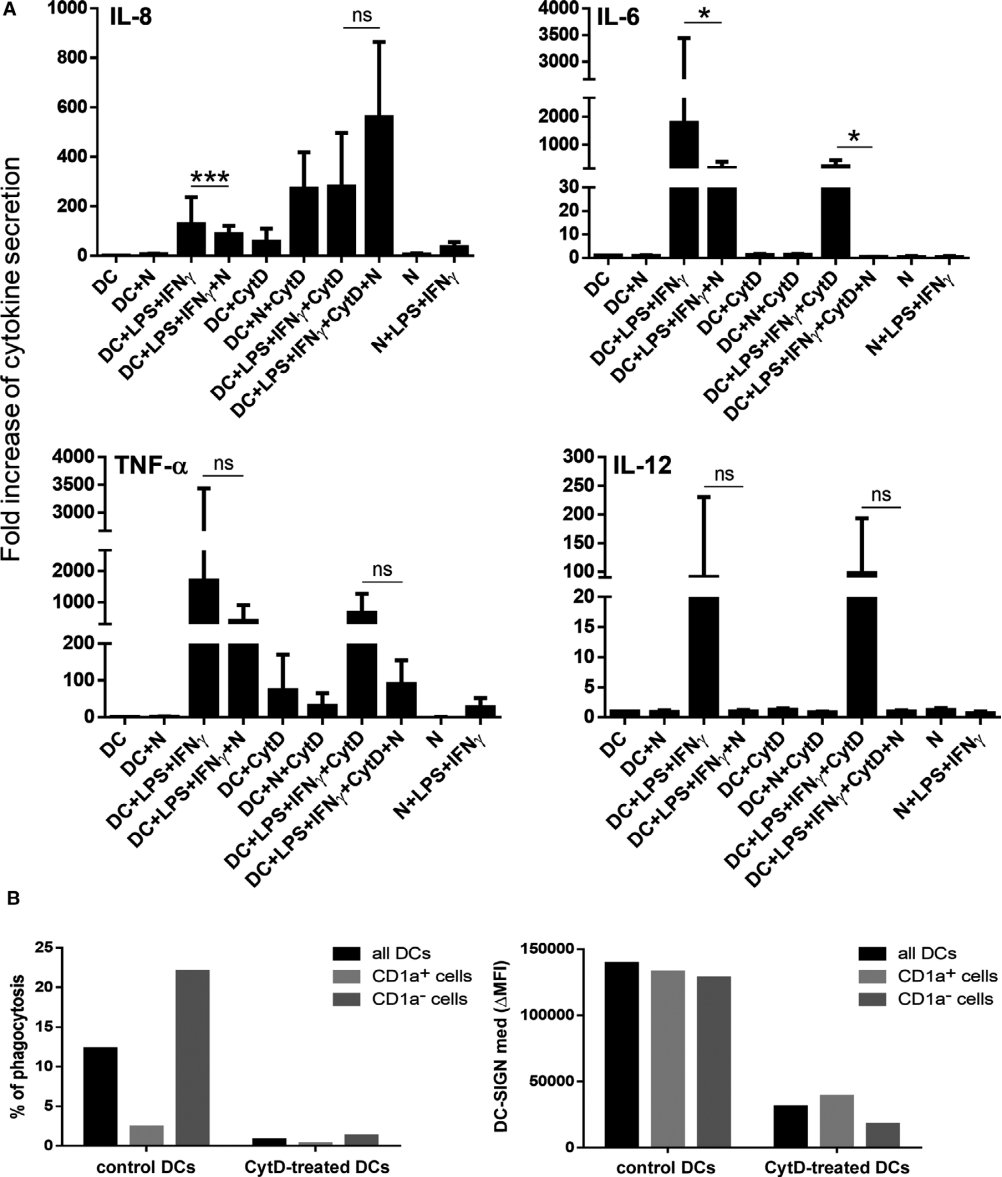
Autologous apoptotic neutrophils inhibit inflammatory mediator production by moDCs in a phagocytosis‐independent manner. Immature moDCs were cocultured with autologous apoptotic neutrophils (N) for 8 h. In some experiments, moDCs were pretreated with 15 µm CytD for 30 min and then cocultured with autologous apoptotic cells in the presence of CytD for 8 h. A fraction of the cells was treated with 0.1 μg·mL^−1^ LPS and 10 ng·mL^−1^ IFN‐γ for another 16 h. Culture supernatants were harvested and stored at −20 °C until cytokine measurements. (A) The concentrations of IL‐8, IL‐6, TNF‐α and IL‐12p70 were measured by using a human inflammatory cytometric bead array. Mediator secretion by treated moDCs is presented as fold increase compared to cytokine production of nonprimed moDCs. The mean ± SD values were calculated from three independent experiments. ANOVA multiparametric test was performed for statistical analysis. ns: not significant, *P < 0.05, ***P < 0.001. (B) Pretreatment with CytD efficiently inhibits phagocytosis (left panel) and downregulates the expression of DC‐SIGN on moDCs (right panel). Phagocytosis and DC‐SIGN expression were investigated by flow cytometry analysis.

To find out whether the inhibition of LPS + IFN‐γ‐induced inflammatory cytokine production by apoptotic cells is phagocytosis‐dependent or not, we pretreated moDCs with CytD, an inhibitor of actin polymerization and phagocytosis, prior to co‐incubation with autologous apoptotic neutrophils. Our results indicate that CytD pretreatment, which prevented phagocytosis (Fig. 3B), did not influence the suppression of IL‐6, TNF‐α and IL‐12, suggesting that the anti‐inflammatory effect of apoptotic neutrophils is independent of phagocytosis (Fig. 3A). However, the LPS + IFN‐γ‐induced production of IL‐8 was not inhibited by the autologous apoptotic neutrophils in this experimental set‐up, which could be explained by the capacity of apoptotic neutrophils to trigger IL‐8 release in CytD‐pretreated moDCs (Fig. 3A). Analysis of the phenotype of moDCs revealed that pretreatment with CytD results in a decrease in the expression of DC‐SIGN, a receptor involved in the engulfment of allogeneic apoptotic neutrophils [9], in both CD1a^+^ and CD1a^−^ subsets (Fig. 3B).

### Coculture with autologous apoptotic neutrophil‐primed moDCs inhibits the production of IL‐17A by T lymphocytes

**Fig. 4:**
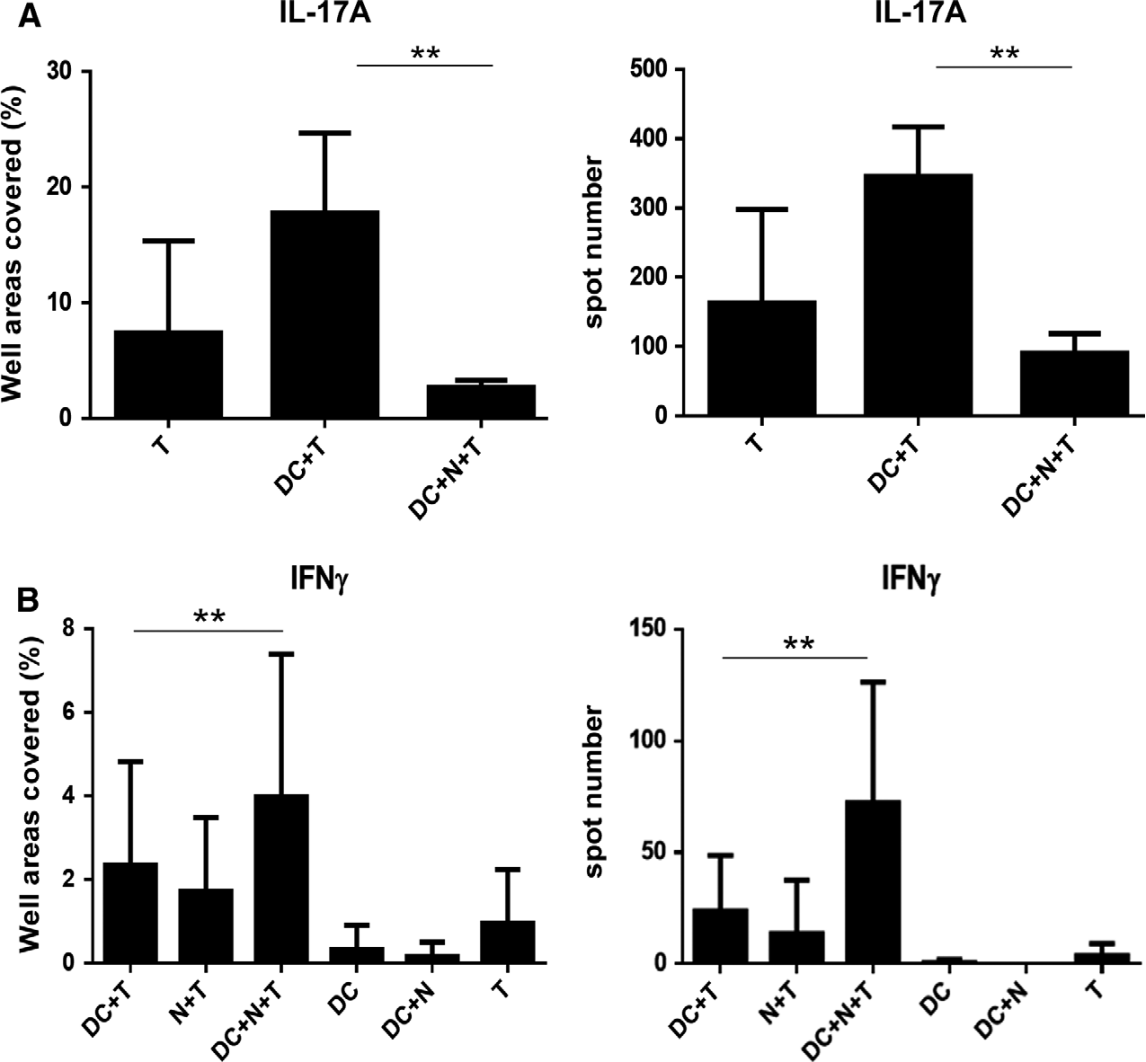
Engulfment of autologous apoptotic neutrophils by moDCs modulates autologous T‐cell responses. Following the capture of autologous apoptotic neutrophils (N), DCs were washed, mixed with autologous lymphocytes (T) at a ratio of 1 : 25 and cocultured for 5 days. The frequency of (A) IL‐17A‐ and (B) IFN‐γ‐secreting cells was determined by ELISPOT assays as described in the Materials and methods. The number of spots was counted, or the area covered by the spots per well was calculated by a computer‐assisted ELISPOT image analyser. The bars represent the mean ± SD of 4–5 parallel measurements performed with the cells from five donors in case of IL‐17A and from nine donors in case of IFN‐γ. **P < 0.01. ANOVA multiparametric test was performed for statistical analysis.

Once the neutrophils are egressed from the bone marrow, their lifespan, migratory potential and further development, as well as the formation of subpopulations, become regulated by a complex cytokine network generated by DCs, macrophages and Th17 lymphocytes. To investigate how the apoptotic neutrophil‐primed moDCs can influence the IL‐17A production by T cells, the immature moDCs were incubated with autologous apoptotic neutrophils for 8 h and then cocultured with autologous lymphocytes for 5 days. In this experimental setting, the number of the IL‐17A‐secreting cells was significantly lower in cocultures containing apoptotic neutrophil‐primed moDCs compared to those containing nonprimed moDCs (Fig. 4A).

### Coculture with autologous apoptotic neutrophil‐primed moDCs enhances IFN‐γ production by T cells

Activated neutrophils can recruit both Th17 and Th1 cells via chemokines [14]; therefore, using the coculture system described above, the effect of apoptotic neutrophil‐primed moDCs on IFN‐γ production by T cells was also examined. As shown in Fig. 4B, the moDCs previously exposed to autologous apoptotic neutrophils have significantly higher potential to increase the number of IFN‐γ‐secreting T lymphocytes compared to those without prior apoptotic cell priming. These results clearly demonstrate the existence of an obvious immunomodulatory potential by autologous apoptotic neutrophils.

## Discussion

Prompt and silent clearance of apoptotic cells seems to be an essential step in the maintenance of tissue homeostasis and supports proper functionality of the immune system [15] acting through dampening inflammation and supporting tolerogenicity to protect self‐components [16]. Neutrophils have been recognized as the first cell types recruited to the site of infection and/or tissue injury, and their timely removal is essential for the resolution of inflammation. Several lines of evidence suggest that neutrophil apoptosis is an important control point in the regulation of acute [15] and chronic inflammatory processes [1, 17] including systemic inflammation in autoimmune diseases.

In this study, we demonstrate that human autologous apoptotic neutrophils are readily engulfed by immature DCs with similar efficiency as allogeneic neutrophils, indicating that this process is not modulated by the origin of the neutrophils. The phagocytosis of autologous apoptotic neutrophils can result in MHC class II‐mediated antigen presentation on the DC surface. However, in the absence of coupled danger or damage signals the apoptotic cells do not acquire immunogenicity due to lack of appropriate costimulation. Consequently, this pathway may lead to tolerance induction instead of provoking inflammation [18, 19, 20, 21]. Although we were unable to detect differences in the ratio of cells showing phagocytic activity in the autologous and the allogeneic settings, the outcome of inflammatory responses turned out to be different. This is remarkable, since the autologous apoptotic neutrophils were able to inhibit the production of inflammatory chemokines and cytokines triggered by additional stimuli, in contrast to the allogeneic setting, where the allogeneic apoptotic neutrophils were unable to inhibit this process [9]. It is worth nothing that the presence of high numbers of apoptotic neutrophils has been reported to induce DC maturation [22]; therefore, the amount of apoptotic bodies taken up by individual cells may have a critical role on DC responses.

During the last decades, numerous anti‐inflammatory mechanisms triggered by apoptotic cells have been described (reviewed in [23]). In line with previous observations that exposure to apoptotic neutrophils may lead to the prevention of DC activation [24], we also found an inhibitory effect of autologous apoptotic neutrophils on the production of cytokines by moDCs upon exposure to inflammatory stimuli. Interestingly, our results indicate that apoptotic autologous neutrophils hamper inflammatory cytokine production by moDCs in a phagocytosis‐independent manner. This finding is in agreement with previous observations that the apoptotic process itself (independent of phagocytosis) can modulate many functions of cells in the microenvironment. Indeed, prior studies have demonstrated that various cells are able to release anti‐inflammatory cytokines during the apoptotic process [25, 26]. Furthermore, it has recently been reported that specific metabolites released from apoptotic cells actively modulate gene expressions and induce an anti‐inflammatory gene signature in the neighbouring cells [27].

In our study, we found that despite their hampered inflammatory cytokine production, autologous apoptotic neutrophil‐primed moDCs can induce autologous T‐cell responses and increase the number of IFN‐γ‐secreting T lymphocytes. Our observation confirms an earlier report showing that DCs loaded with apoptotic cells induced a polyclonal proliferation of autologous naive CD4^+^ T cells even though no phenotypic differences were found between immature and apoptotic cell‐loaded DCs [28]. It was clearly proved that the ability of the apoptotic cell‐primed DCs to generate CD4^+^ T‐cell responses was not due to an increase in the costimulatory capacity of DCs, but was dependent on the classical endolysosomal pathway presentation of apoptotic cell‐derived peptides by MHC‐II molecules. Most of the autologous CD4^+^ T cells stimulated with apoptotic cell‐loaded DCs exhibited suppressive activities; however, a subset of them produced IFN‐γ. The authors supposed that the IFN‐γ production in responding T cells may be a consequence of the presence of apoptotic cells in late apoptosis/secondary necrosis stage among engulfed apoptotic cells [28]. In our experimental set‐up, the possibility of some peculiar danger‐associated molecular pattern molecule release from dying neutrophils can also not be excluded. Importantly, stimulation of macrophages with IFN‐γ enhances their capacity for phagocytosis of apoptotic cells, which is an essential process in tissue homeostasis, immunity and resolution of inflammation [29].

IFN‐γ is a pleiotropic cytokine playing a complex role in the regulation of inflammatory reactions and also in the maintenance of immune homeostasis [30]. Due to its complex modulatory role exerted on various signalling pathways, cell trafficking, APC maturation, antigen presentation and cellular differentiation, it also plays a determining role in disease outcomes [30]. In a series of experiments in murine models, it has been shown that lack of IFN‐γ receptor accelerates, while treatment of animals with IFN‐γ inhibits the development of collagen‐induced arthritis [31, 32, 33, 34]. In a collagen‐induced arthritis model system, it has been proved that neutralization of IL‐17 almost entirely prevents development of the disease and also that IFN‐γ suppresses IL‐17 production [35]. Moreover, it has also been revealed that IFN‐γ inhibits the production of IL‐17A and IL‐17F in a STAT1‐dependent, Tbet‐independent manner [36]. In our experiments, the number of the IL‐17A‐secreting cells was significantly lower in cocultures containing apoptotic neutrophil‐primed moDCs compared to control ones. In the light of literature data and our presented results, we propose that IFN‐γ released from autologous CD4^+^ T cells stimulated with apoptotic cell‐primed DCs is responsible for blocking IL‐17 production.

A form of cell death specific to neutrophils is NETosis. The release of NET results in the emission of alarmins and formation of RNA/DNA complexes serving as costimulatory elements for TLR7/8 and TLR9, thus linking neutrophils to DCs through inducing DC maturation and the promotion of autoimmune diseases [37]. In patients with rheumatoid arthritis [38], TNF‐α and IL‐17A have been shown to promote NET formation contributing to the pathogenesis of the disease. On the other hand, macrophages stimulated with TNF‐α display an impaired ability to ingest apoptotic cells [39], whereas IL‐17A acts as a major orchestrator of sustained neutrophilic mobilization [40]. Here, we report that exposure of immature DCs to autologous apoptotic neutrophils leads to a decreased production of TNF‐α by DCs as well as to an enhanced IFN‐γ and a downregulated IL‐17A release by interacting T cells. This suggests that at physiological circumstances, the balanced level of these cytokines may influence the release of neutrophils, thus preventing the cells from further recruitment, activation and NET formation, all associated with concomitant tissue damage [38, 41, 42].

These data altogether suggest that additional inflammatory stimuli may also modulate the outcome of IFN‐γ signalling. Here, we propose that IFN‐γ, released from DCs engulfing autologous apoptotic neutrophils, has the potential to modulate IL‐17 signalling and thus contribute to the maintenance of a steady‐state condition, or it is involved in supporting physiological autoimmunity, which upon external insults may modify the balance of the immunological network leading to an altered steady state contributing to the development of a pathological outcome.

## Conflict of interest

The authors declare no conflict of interest.

## Author contributions

GEM designed and performed the experiments, analysed the data and wrote the paper. PG and MT performed the experiments, analysed the data and wrote the paper. JH and DH designed and performed the experiments and analysed the data. ÉR, LF and AB designed the research and wrote the paper.

## References

[1] Rosales C (2018) Neutrophil: a cell with many roles in inflammation or several cell types? Front Physiol, 20 :.113 10.3389/fphys.2018.00113 PMC5826082 29515456

[2] Yang D , Chen Q , Chertov O and Oppenheim JJ (2000) Human neutrophil defensins selectively chemoattract naive T and immature dendritic cells. J Leukoc Biol 68, 9–14. 10914484

[3] Puga I , Cols M , Barra CM , He B , Cassis L , Gentile M , Comerma L , Chorny A , Shan M , Xu W *et al* (2011) B cell‐helper neutrophils stimulate the diversification and production of immunoglobulin in the marginal zone of the spleen. Nat Immunol 13, 170–180. 22197976 10.1038/ni.2194 PMC3262910

[4] Scapini P and Cassatella MA (2014) Social networking of human neutrophils within the immune system. Blood 124, 710–719. 24923297 10.1182/blood-2014-03-453217

[5] Yang D , de la Rosa G , Tewary P and Oppenheim JJ (2009) Alarmins link neutrophils and dendritic cells. Trends Immunol 30, 531–537. 19699678 10.1016/j.it.2009.07.004 PMC2767430

[6] Miyasaka K , Hanayama R , Tanaka M and Nagata S (2004) Expression of milk fat globule epidermal growth factor 8 in immature dendritic cells for engulfment of apoptotic cells. Eur J Immunol. 34, 1414–1422. 15114675 10.1002/eji.200424930

[7] Mantovani A , Cassatella MA , Costantini C and Jaillon S (2011) Neutrophils in the activation and regulation of innate and adaptive immunity. Nat Rev Immunol 11, 519–531. 21785456 10.1038/nri3024

[8] Green DR , Ferguson T , Zitvogel L and Kroemer G (2009) Immunogenic and tolerogenic cell death. Nat Rev Immunol 9, 353–363. 19365408 10.1038/nri2545 PMC2818721

[9] Majai G , Gogolak P , Ambrus C , Vereb G , Hodrea J , Fesus L and Rajnavolgyi E (2010) PPARgamma modulated inflammatory response of human dendritic cell subsets to engulfed apoptotic neutrophils. J Leukoc Biol 88, 981–991. 20686116 10.1189/jlb.0310144

[10] Brinkmann V , Reichard U , Goosmann C , Fauler B , Uhlemann Y , Weiss DS , Weinrauch Y and Zychlinsky A (2004) Neutrophil extracellular traps kill bacteria. Science 303, 1532–1535. 15001782 10.1126/science.1092385

[11] Mocsai A (2013) Diverse novel functions of neutrophils in immunity, inflammation, and beyond. J Exp Med 210, 1283–1299. 23825232 10.1084/jem.20122220 PMC3698517

[12] Barrientos L , Bignon A , Gueguen C , de Chaisemartin L , Gorges R , Sandre C , Mascarell L , Balabanian K , Kerdine‐Romer S , Pallardy M *et al* (2014) Neutrophil extracellular traps downregulate lipopolysaccharide‐induced activation of monocyte‐derived dendritic cells. J Immunol 193, 5689–5698. 25339673 10.4049/jimmunol.1400586

[13] Majai G , Sarang Z , Csomos K , Zahuczky G and Fesus L (2007) PPARgamma‐dependent regulation of human macrophages in phagocytosis of apoptotic cells. Eur J Immunol 37, 1343–1354. 17407194 10.1002/eji.200636398

[14] Pelletier M , Maggi L , Micheletti A , Lazzeri E , Tamassia N , Costantini C , Cosmi L , Lunardi C , Annunziato F , Romagnani S (2010) Evidence for a cross‐talk between human neutrophils and Th17 cells. Blood 115, 335–343. 19890092 10.1182/blood-2009-04-216085

[15] Greenlee‐Wacker MC (2016) Clearance of apoptotic neutrophils and resolution of inflammation. Immunol Rev 273, 357–370. 27558346 10.1111/imr.12453 PMC5000862

[16] Henson PM (2005) Dampening inflammation. Nat Immunol 6, 1179–1181. 16369556 10.1038/ni1205-1179

[17] Soehnlein O , Steffens S , Hidalgo A and Weber C (2017) Neutrophils as protagonists and targets in chronic inflammation. Nat Rev Immunol 17, 248–261. 28287106 10.1038/nri.2017.10

[18] Blander JM and Medzhitov R (2006) On regulation of phagosome maturation and antigen presentation. Nat Immunol 7, 1029–1035. 16985500 10.1038/ni1006-1029

[19] Blander JM and Medzhitov R (2004) Regulation of phagosome maturation by signals from toll‐like receptors. Science 304, 1014–1018. 15143282 10.1126/science.1096158

[20] Delamarre L , Pack M , Chang H , Mellman I and Trombetta ES (2005) Differential lysosomal proteolysis in antigen‐presenting cells determines antigen fate. Science 307, 1630–1634. 15761154 10.1126/science.1108003

[21] Erwig LP , McPhilips KA , Wynes MW , Ivetic A , Ridley AJ and Henson PM (2006) Differential regulation of phagosome maturation in macrophages and dendritic cells mediated by Rho GTPases and ezrin‐radixin‐moesin (ERM) proteins. Proc Natl Acad Sci USA 103, 12825–12830. 16908865 10.1073/pnas.0605331103 PMC1568932

[22] Rovere P , Vallinoto C , Bondanza A , Crosti MC , Rescigno M , Ricciardi‐ P , Rugarli C and Manfredi AA (1998) Bystander apoptosis triggers dendritic cell maturation and antigen‐presenting function. J Immunol 161, 4467–4471. 9794367

[23] Szondy Z , Sarang Z , Kiss B , Garabuczi E and Koroskenyi K (2017) Anti‐inflammatory mechanisms triggered by apoptotic cells during their clearance. Front Immunol 8, 909. 28824635 10.3389/fimmu.2017.00909 PMC5539239

[24] Clayton AR , Prue RL , Harper L , Drayson MT and Savage CO (2003) Dendritic cell uptake of human apoptotic and necrotic neutrophils inhibits CD40, CD80, and CD86 expression and reduces allogeneic T cell responses: relevance to systemic vasculitis. Arthritis Rheum 48, 2362–2374. 12905492 10.1002/art.11130

[25] Gao Y , Herndon JM , Zhang H , Griffith TS and Ferguson TA (1998) Antiinflammatory effects of CD95 ligand (FasL)‐induced apoptosis. J Exp Med 188, 887–896. 9730890 10.1084/jem.188.5.887 PMC2213381

[26] Chen W , Frank ME , Jin W and Wahl SM (2001) TGF‐beta released by apoptotic T cells contributes to an immunosuppressive milieu. Immunity 14, 715–725. 11420042 10.1016/s1074-7613(01)00147-9

[27] Medina CB , Mehrotra P , Arandjelovic S , Perrys JSA , Guo YZ , Morioka S , Barron B , Walk SF , Ghesquiere B , Lorenz U *et al* (2020) Metabolites released from apoptotic cells act as tissue messengers. Nature 580, 130–135. 32238926 10.1038/s41586-020-2121-3 PMC7217709

[28] Valente M , Baey C , Louche P , Dutertre CA , Vimeux L , Maranon C , Hosmalin A and Feuillet V (2014) Apoptotic cell capture by DCs induces unexpectedly robust autologous CD4+ T‐cell responses. Eur J Immunol 44, 2274–2286. 24824875 10.1002/eji.201344191

[29] Fernandez‐Boyanapalli R , McPhillips KA , Frasch SC , Janssen WJ , Dinauer MC , Riches DW , Henson PM , Byrne A and Bratton DL (2010) Impaired phagocytosis of apoptotic cells by macrophages in chronic granulomatous disease is reversed by IFN‐gamma in a nitric oxide‐dependent manner. J Immunol 185, 4030–4041. 20805415 10.4049/jimmunol.1001778 PMC4346245

[30] Lees JR (2015) Interferon gamma in autoimmunity: A complicated player on a complex stage. Cytokine 74, 18–26. 25464925 10.1016/j.cyto.2014.10.014

[31] Nakajima H , Takamori H , Hiyama Y and Tsukada W (1990) The effect of treatment with interferon‐gamma on type II collagen‐induced arthritis. Clin Exp Immunol 81, 441–445. 2118846 10.1111/j.1365-2249.1990.tb05353.x PMC1534996

[32] Vermeire K , Heremans H , Vandeputte M , Huang S , Billiau A and Matthys P (1997) Accelerated collagen‐induced arthritis in IFN‐gamma receptor‐deficient mice. J Immunol 158, 5507–5513. 9164974

[33] Manoury‐Schwartz B , Chiocchia G , Bessis N , Abehsira‐Amar O , Batteux F , Muller S , Huang S , Boissier MC and Fournier C (1997) High susceptibility to collagen‐induced arthritis in mice lacking IFN‐gamma receptors. J Immunol 158, 5501–5506. 9164973

[34] Matthys P , Vermeire K , Mitera T , Heremans H , Huang S , Schols D , De Wolf‐Peeters C and Billiau A (1999) Enhanced autoimmune arthritis in IFN‐gamma receptor‐deficient mice is conditioned by mycobacteria in Freund's adjuvant and by increased expansion of Mac‐1+ myeloid cells. J Immunol 163, 3503–3510. 10477624

[35] Chu CQ , Swart D , Alcorn D , Tocker J and Elkon KB (2007) Interferon‐gamma regulates susceptibility to collagen‐induced arthritis through suppression of interleukin‐17. Arthritis Rheum 56, 1145–1151. 17393396 10.1002/art.22453

[36] Yeh WI , McWilliams IL and Harrington LE (2014) IFNgamma inhibits Th17 differentiation and function via Tbet‐dependent and Tbet‐independent mechanisms. J Neuroimmunol 267, 20–27. 24369297 10.1016/j.jneuroim.2013.12.001 PMC4363997

[37] Yang D , Han Z and Oppenheim JJ (2017) Alarmins and immunity. Immunol Rev 280, 41–56. 29027222 10.1111/imr.12577 PMC5699517

[38] Khandpur R , Carmona‐Rivera C , Vivekanandan‐Giri A , Gizinski A , Yalavarthi S , Knight JS , Friday S , Li S , Patel RM , Subramanian V *et al* (2013) NETs are a source of citrullinated autoantigens and stimulate inflammatory responses in rheumatoid arthritis. Sci Transl Med 5, 178ra40. 10.1126/scitranslmed.3005580 PMC3727661 23536012

[39] McPhillips K , Janssen WJ , Ghosh M , Byrne A , Gardai S , Remigio L , Bratton DL , Kang JL and Henson P (2007) TNF‐alpha inhibits macrophage clearance of apoptotic cells via cytosolic phospholipase A2 and oxidant‐dependent mechanisms. J Immunol 178, 8117–8126. 17548650 10.4049/jimmunol.178.12.8117

[40] Laan M , Cui ZH , Hoshino H , Lotvall J , Sjostrand M , Gruenert DC , Skoogh BE and Linden A (1999) Neutrophil recruitment by human IL‐17 via C‐X‐C chemokine release in the airways. J Immunol. 162, 2347–2352. 9973514

[41] von Vietinghoff S and Ley K (2008) Homeostatic regulation of blood neutrophil counts. J Immunol. 181, 5183–5188. 18832668 10.4049/jimmunol.181.8.5183 PMC2745132

[42] Gaffen SL , Jain R , Garg AV and Cua DJ (2014) The IL‐23‐IL‐17 immune axis: from mechanisms to therapeutic testing. Nat Rev Immunol 14, 585–600. 25145755 10.1038/nri3707 PMC4281037

